# Association between risk of malnutrition defined by patient-generated subjective global assessment and adverse outcomes in patients with cancer: a systematic review and meta-analysis

**DOI:** 10.1017/S1368980024000788

**Published:** 2024-03-27

**Authors:** Junfang Zhang, Wei Xu, Heng Zhang, Yu Fan

**Affiliations:** 1 Department of Medical Nutrition, Nanjing Lishui District People’s Hospital, Zhongda Hospital Lishui Branch, Southeast University, Nanjing, China; 2 Institute of Molecular Biology & Translational Medicine, The Affiliated People’s Hospital, Jiangsu University, No. 8 Dianli Road, Zhenjiang, Jiangsu, China; 3 Department of General Surgery, Nanjing Lishui District People’s Hospital, Zhongda Hospital Lishui Branch, Southeast University, No. 86 Chongwen Road, Nanjing, China

**Keywords:** Cancer, Patient-generated subjective global assessment, Overall survival, Postoperative complications, Meta-analysis

## Abstract

**Objective:**

To assess the association between the risk of malnutrition, as estimated by the Patient-Generated Subjective Global Assessment (PG-SGA) numerical scores, and adverse outcomes in oncology patients.

**Design:**

Systematic review and meta-analysis.

**Settings:**

A comprehensive search was conducted in PubMed, Web of Science, Embase, CKNI, VIP, Sinomed and Wanfang databases. Studies that examined the association between the risk of malnutrition, as estimated by the PG-SGA numerical scores, and overall survival (OS) or postoperative complications in oncology patients were included. Patients were classified as low risk (PG-SGA ≤ 3), medium risk (PG-SGA 4–8) and high risk of malnutrition (PG-SGA > 8).

**Subject:**

Nineteen studies reporting on twenty articles (*n* 9286 patients).

**Results:**

The prevalence of medium and high risk of malnutrition ranged from 16·0 % to 71·6 %. A meta-analysis showed that cancer patients with medium and high risk of malnutrition had a poorer OS (adjusted hazard ratios (HR) 1·98; 95 % CI 1·77, 2·21) compared with those with a low risk of malnutrition. Stratified analysis revealed that the pooled HR was 1·55 (95 % CI 1·17, 2·06) for medium risk of malnutrition and 2·65 (95 % CI 1·90, 3·70) for high risk of malnutrition. Additionally, the pooled adjusted OR for postoperative complications was 4·65 (95 % CI 1·61, 13·44) for patients at medium and high risk of malnutrition.

**Conclusions:**

The presence of medium and high risk of malnutrition, as estimated by the PG-SGA numerical scores, is significantly linked to poorer OS and an increased risk of postoperative complications in oncology patients.

Cancer remains a significant public health concern, with an estimated 19·3 million new cases and 10·0 million cancer-related deaths in 2020^(1)^. Despite advancements in diagnostic techniques and therapeutic strategies, the long-term prognosis for patients with advanced cancer remains poor^(2)^. Therefore, there is an urgent need to enhance the prognostic assessment of cancer patients.

Malnutrition is a prevalent issue among cancer patients^(3)^. The European Society for Clinical Nutrition and Metabolism guidelines on nutrition strongly recommend screening the nutritional status of all cancer patients^(4)^. Malnutrition in cancer patients has been linked to increased postoperative complications, prolonged hospitalisation, reduced tolerance to treatment, worsened survival and lower quality of life^(5)^. Therefore, nutritional evaluation in such patients is of paramount importance.

Several screening and assessment tools have been developed to evaluate the nutritional status of cancer patients. However, there is no universally accepted standard for defining malnutrition in this population^(6,7)^. Among these tools, the Nutritional Risk Screening-2002 and the Patient-Generated Subjective Global Assessment (PG-SGA) were the most commonly used for nutritional evaluation in adults with cancer^(8)^. The PG-SGA numerical scores have been used internationally as the reference method for risk screening, assessment, monitoring and triaging for interventions in patients with cancer^(9)^. This tool includes both patient-reported (self-reported weight change, changes in food intake, presence of nutrition impact symptoms and activities and function) and clinician-assessed (scoring weight loss, physical examination, metabolic stress and disease and its relation to nutritional requirements) components. A higher PG-SGA score indicates a higher risk of malnutrition. Patients were classified as low risk (PG-SGA ≤ 3), medium risk (PG-SGA 4–8) and high risk of malnutrition (PG-SGA > 8). The prognostic significance of this nutritional tool has been widely studied in cancer patients^(10–16)^. However, the existing studies have reported inconsistent findings regarding the association between the risk of malnutrition, as estimated by the PG-SGA numerical scores, and overall survival (OS)^(17,18)^. Furthermore, conflicting results have been reported regarding the prognostic significance of medium risk of malnutrition in these patients^(11,17,18)^. Therefore, we conducted this meta-analysis to evaluate the prognostic utility of malnutrition risk, as estimated by the PG-SGA numerical scores, in cancer patients.

## Methods

### Search strategy

The current systematic review/meta-analysis was reported in accordance with the guidelines of Preferred Reporting Items for Systematic Reviews and Meta-Analyses^(19)^. A systematic search was performed in multiple databases, including PubMed, Web of Science, Embase, CKNI, VIP, Sinomed and Wanfang databases through 28 March 2023, without any language restrictions. Two authors independently searched the English literature using the following keywords: ‘Patient-Generated Subjective Global Assessment’ OR ‘PG-SGA’ AND ‘cancer’ OR ‘tumor’ OR ‘malignancy’ OR ‘carcinoma’ OR ‘neoplasms’ AND ‘complication’ OR ‘survival’ OR ‘mortality’ OR ‘death’. For Chinese literature, the keywords used were: ‘Zhong liu’ AND ‘ai’ AND ‘huan zhe zhu guan zheng ti ping gu’ AND ‘sheng cun’ OR ‘si wang’ AND ‘bing fa zheng’. The detailed search strategy is presented in see online supplementary material, Supplemental Text S1. In addition, the reference lists of retrieved studies and pertinent reviews were manually searched to identify additional studies.

### Study selection

Two authors independently selected studies based on the following criteria for inclusion: (1) population: adult patients diagnosed with cancer; (2) comparator: risk of malnutrition, as estimated using the PG-SGA numerical scores; (3) comparison: medium and high risk of malnutrition (PG-SGA score >4) *v*. low risk of malnutrition (PG-SGA score ≤3); (4) outcomes of interest: OS or postoperative complications defined by the Clavien–Dindo classification system; (5) type of study: either retrospective or prospective cohort and (6) reported a multivariable adjusted hazard ratio (HR) or OR with 95 % CI for the abovementioned outcomes. In cases where multiple publications were derived from the same population, only the study with the most comprehensive information was included. Articles from the same cohort but with specific type of cancer were included in subgroup analysis. The criteria for exclusion were (1) risk of malnutrition was estimated using other nutritional assessment tools; (2) lack of outcomes of interest; (3) reported of the unadjusted risk estimate; (4) not selecting the low risk of malnutrition (PG-SGA score ≤3) as the reference group; (5) overlapping participants with other articles and (6) inclusion of meeting abstracts, reviews or cross-sectional studies.

### Data extraction and Quality assessment

Data extracted from the individual studies included: first author’s name, publication year, origin of patients, study design, cancer type, sample size, proportion of male participants, age at enrollment, assessing risk of malnutrition, risk of malnutrition prevalence, outcome measures, length of follow-up, fully adjusted relative risk and adjustment for variables. To assess the methodological quality of the included studies, a nine-point Newcastle-Ottawa Scale was used^(20)^. The overall quality was categorised as low (<4 points), moderate (4-6 points) or high (≥7 points), respectively. Two independent authors performed data extraction and quality assessment. Any disagreements were resolved through consensus or discussion with the corresponding author.

### Statistical analyses

All meta-analyses were undertaken using Stata 12·0 (Stata Corporation). For OS (time-to-event data), the prognostic value was expressed by pooling the adjusted HR with 95 % CI for the medium and high risk of malnutrition *v*. low risk of malnutrition group. The pooled adjusted OR with 95 % CI was used to summarise the association between risk of malnutrition with postoperative complications. Study heterogeneity was assessed using the *I* ^2^ statistic and Cochran’s *Q* test. An I^2^ statistics of <50 % and/or a *P* value >0·10 for the Cochran *Q* test indicated no significant heterogeneity, and a fixed-effect model was used for meta-analysis. If significant heterogeneity was present, a random-effects model was used. Sensitivity analysis was carried out by repeating the analyses after removing one study at a time. Subgroup analyses were undertaken based on study design (retrospective or prospective), cancer type (all types of cancer or gastrointestinal cancer or specific cancer), number of patients (≥500 or <500), age at enrollment (≥60 years or <60 years), geographical region (East Asia or other areas), degree of risk of malnutrition (medium or high) and length of follow-up (≥1 year or < 1 year). Publication bias was evaluated using the Begg’s test^(21)^ and Egger’s test^(22)^. To investigate the potential influence of publication bias, a trim-and-fill analysis was performed.

## Results

### Search results and studies’ characteristics

**Fig. 1:**
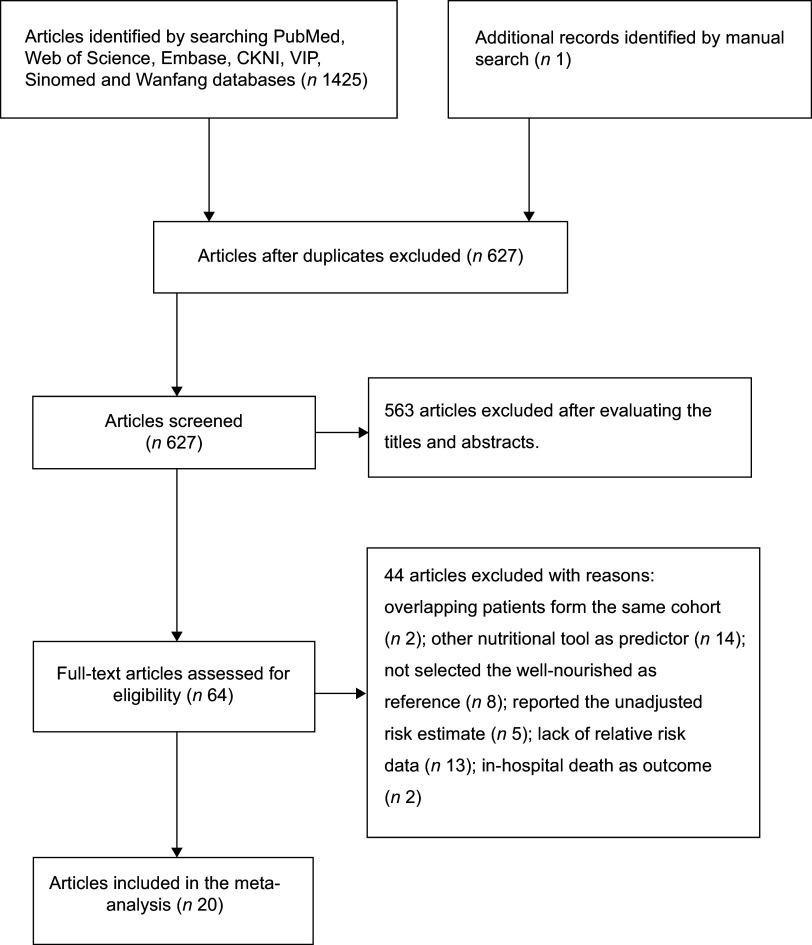
Flow chart showing the process of study selection

Figure 1 summarises the process of study selection. Out of 1425 potentially relevant articles identified in the initial literature search, 627 remained after excluding duplicates. After evaluating the titles and abstracts, 562 articles were subsequently excluded. Sixty-four articles were retrieved for full-text assessment. After applying the predefined inclusion and exclusion criteria, nineteen studies reporting on twenty articles^(10–18,23–32)^ were finally included in this meta-analysis. Among these, Zhang^(27)^ and Ruan^(29)^ reported on all types of cancer and a colorectal cancer subgroup from the same cohort.

The descriptive characteristics of the eligible studies are shown in Table 1. These studies were published from 2015 to 2023 and originated from Brazil, Chile, Australia, South Africa, France, Korea, Iran, Taiwan and China. Eight articles^(10,12,18,23,26,28,30,31)^ adopted the prospective designs, while the remaining articles used retrospective designs. Four articles^(10,14,26,27)^ included all types of cancer, while the others focused on specific types such as oesophageal cancer, gastric cancer, colorectal cancer, hepatocellular carcinoma, gynaecologic cancer, nasopharyngeal carcinoma, oral cancer, head and neck cancer and multiple myeloma. The included studies enrolled a total of 9286 patients with cancer, with sample sizes ranging from 70 to 3547 cases. The prevalence of medium and high risk of malnutrition, as estimated by the PG-SGA numerical scores, varied between 16·0 %^(14)^ and 71·6 %^(28)^. The quality of the studies included is summarised in see online supplementary material, Supplemental Table S1. According to the Newcastle-Ottawa Scale criteria, two articles^(14,31)^ were classified as moderate quality, while the rest were deemed to be of high quality.

**Table 1:** Main characteristic of the included studies

Author/year	Region	Study design	Cancer type	Sample size	% men	Age (years)	Median	Medium/high malnutrition risk (%)	Outcomes		Follow-up	Adjusted variables
									HR	95 % CI		
Tan 2015^(10)^	Australia	P	Advanced cancer	114	61	Median 62		57·9 %	OS 1·79*	1·18, 2·72	13·9 months	Age, sex, weight loss, modified Glasgow Prognostic Score, chemotherapy dose
									1·61	0·96, 2·70 M		
									2·19	1·08, 4·46 H		
Rodrigues 2015^(17)^	Brazil	R	Gynecologic cancer	228	0	55·3	14·9	62·3 %	OS 1·70*	0·93, 3·10	>1 year	Tumour site, cancer stage, elective surgery, complications, Avaliacao Subjetiva Global Produzida Pelo Proprio Paciente
									0·90	0·25, 3·20 M		
									2·04	1·03, 4·05 H		
Kim 2017^(11)^	Korea	R	Multiple myeloma	895	53·7	59	22–85	71·3 %	OS 1·84*	1·21, 2·82	30 months	Age, ISS stage, BMI, LDH, calcium, creatinine, albumin, β-2 microglobulin, cytogenetics, FISH, treatment with novel agents, ASCT, chemotherapy
									1·48	0·83, 2·67 M		
									2·35	1·27, 4·33 H		
Barao 2017^(18)^	Brazil	P	CRC	250	51·6	70·9	7·49	39·6 %	OS 1·61*	0·91, 2·86	10·8 months	Phase angle, BMI, Eastern Cooperative Oncology Group Performance status, TNM stage
									0·95	0·50, 1·81 M		
									12·0	3·44, 42·2 H		
Maurício 2018^(23)^	Brazil	P	CRC	82	46·4	61·6	13·1	52·4 %	Complications 2·08	1·06, 4·06	–	Cancer stage, blood transfusion
Huang 2019^(24)^	Taiwan	R	HCC	287	74·9	63	56–70	33·4 %	Complications 9·85	5·15, 18·86	–	Age, extent of operation, transfusion, blood loss, comorbidity
Gallois 2019^(12)^	France	P	Metastatic CRC	168	56	70	33–93	43 %	OS 2·6	1·3, 5·3	23 months	Multivariate analysis
Tsai 2020^(25)^	Taiwan	R	Oral cancer	70	92·9	72	68–77	62·8 %	Complications 4·93	1·52, 16·03	–	Age, BMI, TNM stage, NLR, comorbidity
Fang 2020^(13)^	China	R	HCC	245	75·5	62·6	9·7	60 %	OS 1·98	1·31, 3·00	30 months	Age, cirrhosis, cancer stage,
De Groot 2020^(14)^	Australia	R	All cancers	246	26	61·9	13·1	16 %	OS 10·37	3·75, 28·68	1 year	Age, sex, BMI, types of cancer
Chen 2021^(15)^	Taiwan	R	Esophageal cancer	340	NP	55·6–57·3	Median	50 %	OS 1·55	1·13, 2·15	8 years	Age, clinical stage, treatment, response to concurrent chemoradiotherapy
Findlay 2021^(16)^	Australia	R	HNC	277	78	60	13	24·9 %	OS 3·03	1·87, 4·93	8 years	Age, sex, ethnicity, performance status, disease stage, CCI
									2·57	1·45, 4·55 M		
									3·19	1·44, 7·07 H		
Von Geldern 2021^(26)^	Chile	P	All cancer	103	44·7	54·9	13·5	54 %	OS 2·42	1·58, 3·72	38 months	Age, sex, TNM stage, sarcopenia
Zhang 2021^(27)^	China	R	All cancer	3777	58·1	56·4	12·1	63·7 %	OS 1·88	1·53, 2·31	1 year	Age, sex, primary tumour types, TNM stage, nutrition treatment, KPS, albumin, NLR, haemoglobin, smoking, alcohol, surgery, chemotherapy, radiotherapy
Nikniaz 2022^(28)^	Iran	P	Gastric cancer	302	69·2	67·4	12	71·6 %	OS 2·04	1·16, 3·58	30 months	Age, TNM stage, BMI, treatment
Ruan 2022^(29)^	China	R	CRC	1358	59·6	60	52–67	61·93 %	OS 1·42	1·14, 1·77	5 years	Age, sex, comorbid disease, tumour site, stage, metastasis, surgery, radiotherapy, chemotherapy and immunotherapy
de Sousa 2022^(30)^	Brazil	P	Gastric cancer, CRC	178	50·6	60·8	12·6	48 %	OS 2·9	1·5, 5·9	12·3 months	Age, TNM stage, type of treatment, site of cancer
Argefa 2022^(31)^	South Africa	P	Cervical cancer	175	0	>18		17·7 %	OS 3·12	1·23, 7·86	8·5 months	Age, parity, stage of cancer, HIV status, BMI
da Silva Couto 2023^(32)^	Brazil	R	CRC	191	57·6	60·5	11·3	32 %	OS 1·65	1·05, 2·60	5 years	Age, sex, performance status, cancer stage, treatment

P, prospective; OS, overall survival; R, retrospective; ISS, International Staging System; LDH, lactate hydrogenase; FISH, fluorescence in situ hybridization; ASCT, autologous stem cell transplantation; CRC, colorectal cancer; TNM, tumour node metastasis; HCC, hepatocellular carcinoma; NLR, neutrophil-to-lymphocyte ratio; NPC, nasopharyngeal carcinoma; HNC, head and neck cancer; CCI, Charlson Comorbidity Index; KPS, Karnofsky performance status; NLR, neutrophil-to-lymphocyte ratio.

* Results pooling from the sub-group using a fixed-effect model.

### Overall survival

**Fig. 2:**
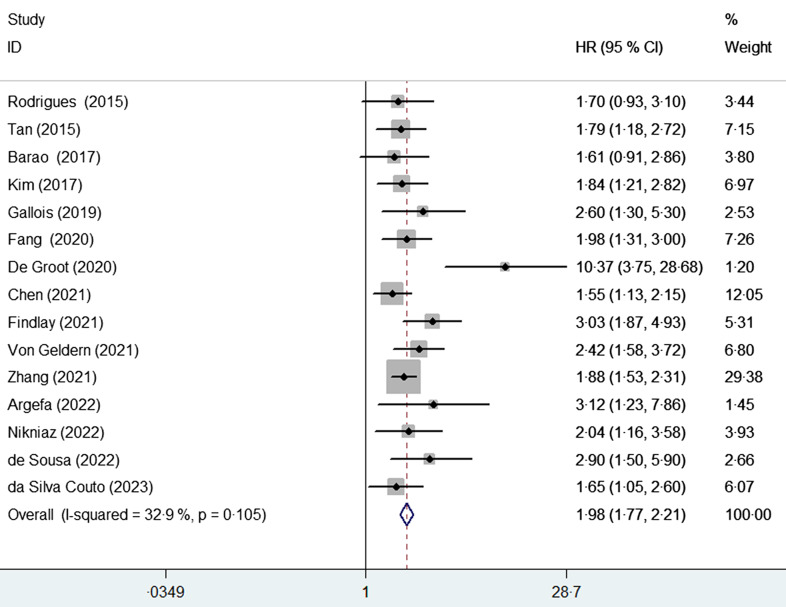
Pooled adjusted hazard ratio with 95 % CI of overall survival for medium and high risk of malnutrition *v*. those with low risk of malnutrition

**Fig. 3:**
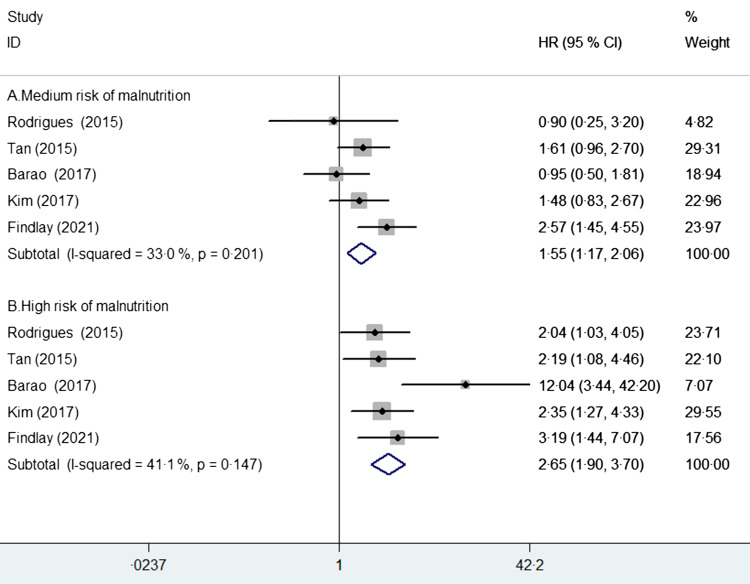
Sub-group analysis on overall survival based on the medium (A) and high (B) risk of malnutrition, respectively

Fifteen studies^(10–18,26–28,30–32)^ examined the association between risk of malnutrition as measured by the PG-SGA and OS. As shown in Fig. 2, medium and high risk of malnutrition was associated with a significantly worse OS (HR 1·98; 95 % CI 1·77, 2·21) compared with those with low risk of malnutrition, without significant heterogeneity (*I* ^2^ = ^(27,28,30–32)^32·9 %; *P* = 0·105). Sensitivity analysis demonstrated the credibility of the original risk summary. Sub-group analysis based on the degree of risk of malnutrition showed that the pooled HR of OS was 1·55 (95 % CI 1·17, 2·06) for medium risk of malnutrition and 2·65 (95 % CI 1·90, 3·70) for high risk of malnutrition, respectively (Fig. 3). Moreover, medium and high risk of malnutrition significantly predicted OS in each predefined sub-group (Table 2). However, Begg’s test (*P* = 0·023) and Egger’s test (*P* = 0·027) suggested the presence of publication bias. Despite this, the pooled HR for OS remained statistically significant (HR 1·88; 95 % CI 1·24, 2·84) after imputing three potentially missing studies using the trim-and-fill analysis (see online supplementary material, Fig. S1).

**Table 2:** Results of sub-group analysis on overall survival

Sub-group	No. of studies	Pooled hazard ratio	95 % confidence intervals	Heterogeneity between studies
Study design				
Prospective	7	2·15	1·74, 2·65	*P* = 0·720; *I* ^2^ = 0·0 %
Retrospective	8	1·92	1·68, 2·19	*P* = 0·022; *I* ^2^ = 57·2 %
Cancer types				
Gastrointestinal cancer	7	1·61	1·39, 1·87	*P* = 0·358; *I* ^2^ = 9·3 %
Colorectal cancer	4	1·53	1·28, 1·84	*P* = 0·429; *I* ^2^ = 0·0 %
Gynecologic cancer	2	2·04	1·23, 3·37	*P* = 0·282; *I* ^2^ = 13·7 %
Geographical region				
Asia	6	1·82	1·59, 2·09	*P* = 0·927; *I* ^2^ = 0·0 %
Others	9	2·34	1·93, 2·84	*P* = 0·056; *I* ^2^ = 47·3 %
Number of patients				
<500	13	2·04	1·78, 2·35	*P* = 0·061; *I* ^2^ = 40·9 %
≥500	2	1·87	1·56, 2·25	*P* = 0·929; *I* ^2^ = 0·0 %
Mean/median age				
<60 years	6	1·87	1·62, 2·16	*P* = 0·553; *I* ^2^ = 0·0 %
≥60 years	9	2·16	1·81, 2·58	*P* = 0·053; *I* ^2^ = 47·9 %
Follow-up duration				
< 1 year	2	1·93	1·19, 3·15	*P* = 0·234; *I* ^2^ = 29·4 %
≥1 year	13	1·98	1·77, 2·22	*P* = 0·078; *I* ^2^ = 38·3 %

### Postoperative complications

**Fig. 4:**
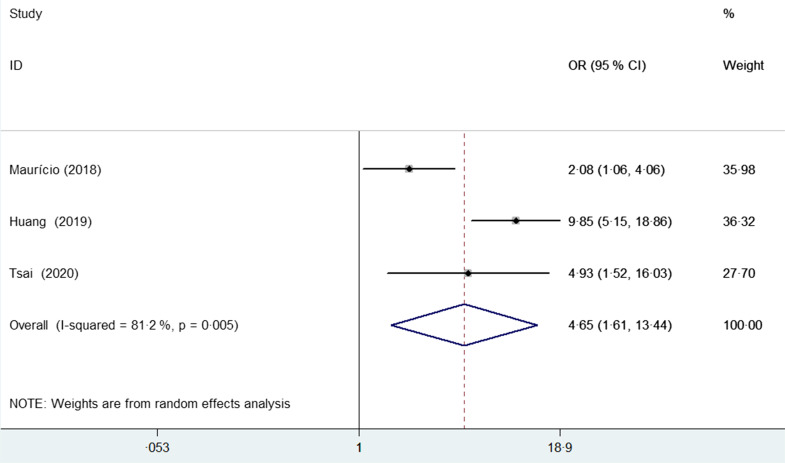
Pooled adjusted OR with 95 % CI of postoperative complications for medium and high risk of malnutrition *v*. those with low risk of malnutrition

Three studies^(23–25)^ examined the association between risk of malnutrition, as estimated by the PG-SGA, and postoperative complications. As shown in Fig. 4, medium and high risk of malnutrition was associated with an increased risk of postoperative complications (OR 4·65; 95 % CI 1·61, 13·44) compared with those with low risk of malnutrition, with significant heterogeneity (*I* ^2^ = 81·2 %; *P* = 0·005). Sensitivity analysis confirmed the robustness of the originally statistical significance of the pooled risk summary.

## Discussion

This systematic review and meta-analysis first evaluated the association between the risk of malnutrition, as estimated by the PG-SGA numerical scores, and adverse outcomes in cancer patients. Overall, the studies included in this analysis were of high methodological quality. Our meta-analysis revealed that the medium and high risk of malnutrition, as measured by the PG-SGA numerical scores, was significantly associated with poorer OS in cancer patients. Specifically, cancer patients with a medium to high risk of malnutrition had approximately twice the risk of reduced OS compared with those with a low risk of malnutrition. The association was even stronger in high-risk malnourished patients (HR 2·65) compared with medium-risk malnourished patients (HR 1·55). Further stratified analysis indicated that medium and high risk of malnutrition consistently correlated with poorer OS, irrespective of study design, cancer type, sample size, degree of malnutrition risk and length of follow-up.

In addition to OS, the risk of malnutrition, as measured by the PG-SGA numerical scores, was found to be linked to a higher risk of postoperative complications. According to our meta-analysis, cancer patients with medium and high risk of malnutrition had a 4·65-fold increased risk of postoperative complications. A randomized, single-blind clinical trial also demonstrated that medium risk of malnutrition was associated with a higher risk of complications in patients with head and neck cancer^(33)^. These complications can result in higher mortality and morbidity rates among cancer patients undergoing surgery. Furthermore, serious postoperative complications can also prolong hospital stays. These is evident in patients with risk of malnutrition and head and neck cancer^(16)^, colorectal cancer^(34)^ and gynecological cancer^(35)^.

There is no consensus on which specific nutritional assessment tool best predicts survival outcomes in cancer patients. Several systematic reviews and meta-analyses have evaluated the value of malnutrition in predicting OS in cancer patients, including the Controlling Nutritional Status (CONUT) score^(36)^, Prognostic Nutritional Index (PNI)^(37)^, Geriatric Nutritional Risk Index (GNRI)^(38)^ and Global Leadership Initiative on Malnutrition (GLIM)^(39)^. Interestingly, the relative risk magnitude for OS was similar in GNRI (HR 1·95), PNI (HR 1·89) and GLIM (HR 1·90). This indicates that the risk of malnutrition, as estimated by the PG-SGA numerical scores, has similar prognostic power in patients with cancer. However, the prognostic value was stronger for PG-SGA-defined high risk of malnutrition (HR 2·65) in the current study compared with the previous GLIM-defined severe malnutrition (HR 1·68). One possible explanation for this finding may be the higher sensitivity and specificity of the PG-SGA numerical scores compared with the GLIM-defined malnutrition^(29)^. It is important to note that these findings were based on indirect comparisons. Further research is needed to fully understand the prognostic significance of malnutrition in various types of cancer, and it may be beneficial to analyze data separately for each specific cancer type.

The Oncology Nutrition Dietetic Practice Group of the American Dietetic Association uses the PG-SGA as the standard for nutritional evaluation in cancer patients^(40)^. Compared with other nutritional assessment tools, the PG-SGA criteria enable a more objective evaluation of nutritional status and the identification of nutritional impact symptoms. Unlike other tools, the PG-SGA relies less on subjective responses from individuals. The PG-SGA numerical scores can indicate changes over time. A study found that for every point increase in PG-SGA score, there was a 4 % higher risk of death in cancer patients receiving a cachexia support service^(41)^. In patients with nasopharyngeal carcinoma, a multivariate-adjusted Cox regression analysis showed that each point increase in PG-SGA score was associated with a 7 % decrease in OS^(42)^. These findings further support the prognostic significance of the PG-SGA numerical scores in cancer patients.

The present study has important implications for clinical practice. The PG-SGA can serve as a promising nutritional screening tool and prognostic indicator of patients’ survival in patients with various types of cancer. For cancer patients at high risk of malnutrition, the PG-SGA numerical scores may provide more accurate prognostic information compared with other nutritional assessment tools. The clinical relevance of the PG-SGA numerical scores lies in its ability to identify patients who are at risk of malnutrition. By identifying and addressing nutritional challenges early, healthcare professionals can implement timely interventions to improve nutritional status and potentially enhance treatment outcomes. Furthermore, regular reassessment using the PG-SGA enables healthcare professionals to track changes in nutritional status and adjust interventions accordingly. However, further research is needed to explore the prognostic value of PG-SGA-defined the risk of malnutrition, particularly through separate analysis of primary cancer types.

Several limitations need to be mentioned in our study. First, the inclusion of retrospective studies in the meta-analysis may have been influenced by their inherent selection bias. Second, there was significant heterogeneity in certain sub-group analyses. This variation could potentially be attributed to differences in clinicopathologic characteristics, types of cancer, study design and follow-up intervals. Third, the results of Begg’s and Egger’s tests revealed the presence of publication bias. However, the trim-and-fill analysis showed that the prognostic value of PG-SGA-defined the risk of malnutrition may have been only slightly overestimated. Finally, this systematic review and meta-analysis has not been prospectively registered in PROSPERO or any other international databases prior to its publication.

### Conclusions

This systematic review/meta-analysis provides *evidence* that medium and high risk of malnutrition, as estimated by the PG-SGA numerical scores, is significantly linked to poorer OS and an increased risk of postoperative complications in oncology patients. Evaluating the numerical scores of the PG-SGA numerical scores can offer crucial prognostic information for these patients.

## Supporting information

Zhang et al. supplementary material 1 Zhang et al. supplementary material

Zhang et al. supplementary material 2 Zhang et al. supplementary material

Zhang et al. supplementary material 3 Zhang et al. supplementary material

## Supplementary material

For supplementary material accompanying this paper visit https://doi.org/10.1017/S1368980024000788
