# Supplementary material for: Association between risk of malnutrition defined by patient-generated subjective global assessment and adverse outcomes in patients with cancer: a systematic review and meta-analysis
Source: Public Health Nutr. 2024 Mar 27;27(1):e105. doi: 10.1017/S1368980024000788 (PMC11010050; doi:10.1017/S1368980024000788)

Figure S1 Trim-and-filled analysis showing the association of malnutrition with overall survival. The circles alone are real studies and the circles enclosed in boxes are ‘filled’ studies.


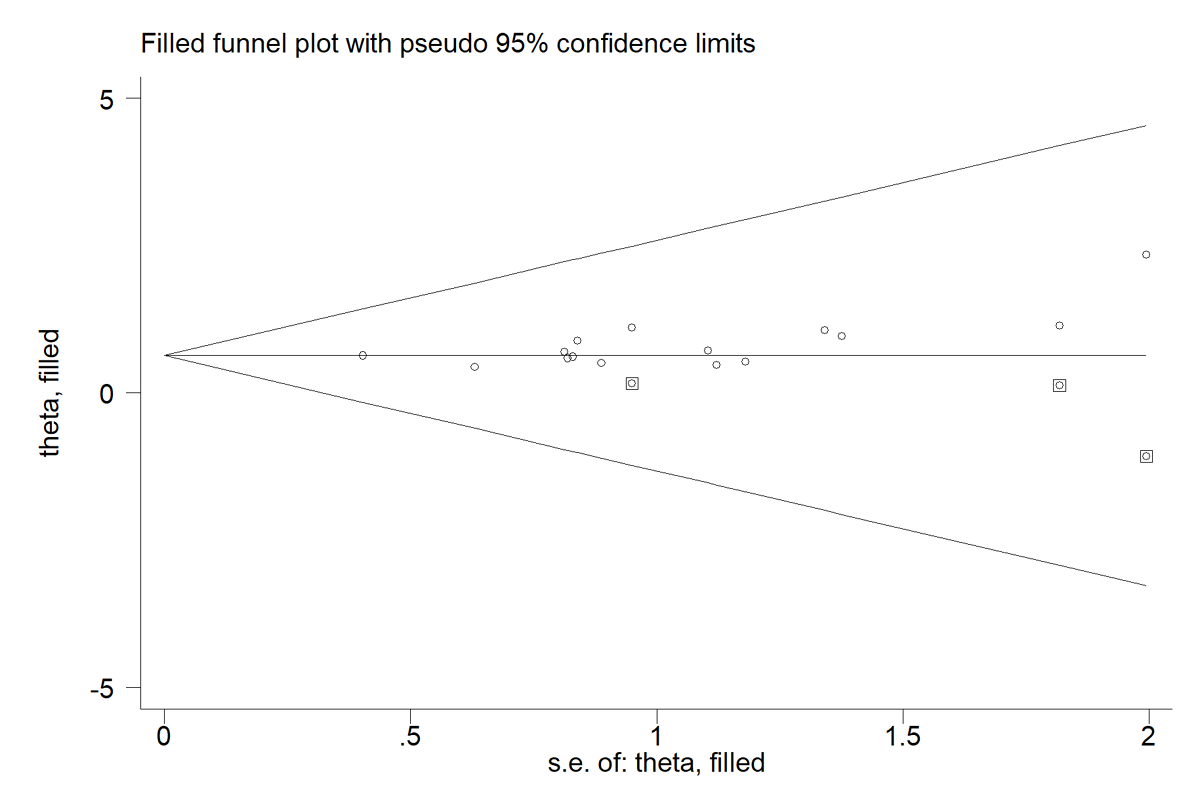

Supplement: Zhang et al. supplementary material 1 — Zhang et al. supplementary material [file S1368980024000788sup001.docx]
