# Supplementary material for: Association between risk of malnutrition defined by patient-generated subjective global assessment and adverse outcomes in patients with cancer: a systematic review and meta-analysis
Source: Public Health Nutr. 2024 Mar 27;27(1):e105. doi: 10.1017/S1368980024000788 (PMC11010050; doi:10.1017/S1368980024000788)
Supplement: Zhang et al. supplementary material 2 — Zhang et al. supplementary material [file S1368980024000788sup002.docx]

**Supplemental Text S1 – Search strategy**

**Limits: Publications until** March 28, 2023

| **1. PubMed Search** | Query | Items found |
| --- | --- | --- |
| #1 | Search: **((Patient-Generated Subjective Global Assessment) OR (PG-SGA)) OR (PGSGA)** | 604 |
| #2 | Search: **((((cancer) OR (tumor)) OR (carcinoma)) OR (malignancy)) OR (neoplasms)** | 5,512,351 |
| #3 | Search: **(((survival) OR (mortality)) OR (death)) OR (complications)** | 6,354,201 |
| #4 | Search: **((#1) AND (#2)) AND (#3)** | 312 |
|  |  |  |

| **2. Embase Search** | Query | Items found |
| --- | --- | --- |
| #1 | Search 'patient-generated subjective global assessment' OR ('patient generated' AND subjective AND ('global'/exp OR global) AND ('assessment'/exp OR assessment)) | 920 |
| #2 | Search 'PG-SGA' | 828 |
| #3 | Search 'PGSGA' | 860 |
| #4 | Search #1 OR #2 OR #3 | 1,098 |
| #5 | Search 'tumor'/exp OR tumor | 6,650,857 |
| #6 | Search 'cancer'/exp OR cancer | 6,055,737 |
| #7 | Search 'carcinoma'/exp OR carcinoma | 1,703,585 |
| #8 | Search 'malignancy'/exp OR malignancy | 316,906 |
| #9 | Search 'neoplasms'/exp OR neoplasms | 5,919,940 |
| #10 | Search #5 OR #6 OR #7 OR #8 OR #9 | 7,658,393 |
| #11 | Search 'survival'/exp OR survival | 2,151,681 |
| #12 | Search 'mortality'/exp OR mortality | 1,877,470 |
| #13 | Search 'death'/exp OR death | 1,768,810 |
| #14 | Search 'complications'/exp OR complications | 2,404,932 |
| #15 | Search #11 OR #12 OR #13 OR #14 | 6,474,821 |
| #16 | Search #4 AND #10 AND #15 | 353 |
| #17 | Search #16 AND ('article'/it OR 'article in press'/it) | 203 |

| **3.** **Web of Science** | Query | Items found |
| --- | --- | --- |
| #1 | Search “Patient-Generated Subjective Global Assessment” OR “PG-SGA” OR “PGSGA” (All fields) | 646 |
| #2 | Search "tumor" OR "cancer" OR "carcinoma" OR "malignancy" OR “neoplasms” (Search within all fields of #1) | 525 |
| #3 | Search "survival" OR "mortality" OR "death" OR “complication” (Search within all fields of #2) | 247 |

| **4. China National Knowledge Infrastructure** | Query | Items found |
| --- | --- | --- |
| #1 | Search ([Title/keyword/abstract] = “肿瘤“ OR ”癌“ OR “恶性疾病” | 1,070,931 |
| #2 | Search “患者主观整体评估[All text]“ (Search within all fields of #1) | 237 |
| #3 | Search “生存 [All text]“ OR ”死亡[All text]“ OR ”并发症 [All text]“(Search within all fields of #2) | **222** |

| **5. VIP Databases** | Query | Items found |
| --- | --- | --- |
| #1 | Search ([Title/keyword] = “肿瘤” OR “癌” OR “恶性疾病”) | 1,139,095 |
| #2 | Search “患者主观整体评估[Abstract]”  (Search within all fields of #1) | **202** |

**6. Wanfang Databases**

Search (“肿瘤” OR “癌” OR “恶性疾病”) AND (“患者主观整体评估”) **131 Items**

**7. Chinese Biomedical Literature Database (Sinomed)**

Search (“肿瘤” OR “癌” OR “恶性疾病”) AND (“患者主观整体评估”) **108 Items**
