# Supplementary material for: Association between risk of malnutrition defined by patient-generated subjective global assessment and adverse outcomes in patients with cancer: a systematic review and meta-analysis
Source: Public Health Nutr. 2024 Mar 27;27(1):e105. doi: 10.1017/S1368980024000788 (PMC11010050; doi:10.1017/S1368980024000788)
Supplement: Zhang et al. supplementary material 3 — Zhang et al. supplementary material [file S1368980024000788sup003.doc]

~~Supplemental Table S1 Methodological quality assessment of the included studies using the Newcastle-Ottawa Scale~~

| Author/Year | Representativeness of the exposed cohort | Selection of the non exposed cohort | Ascertainment of exposure | Demonstration that outcome was not present at study start | Comparability of cohorts based on the design or analysis | Assessment of outcome | Enough follow-up periods (≥1 year) | Adequacy of follow-up of cohorts | Total scores |
| --- | --- | --- | --- | --- | --- | --- | --- | --- | --- |
| Tan 2015 ^(10)^ |  | ★ | ★ | ★ | ★ | ★ | ★ | ★ | 7 |
| Rodrigues 2015 ^(17)^ |  | ★ | ★ | ★ | ★★ | ★ | ★ | ★ | 8 |
| Kim 2017 ^(11)^ | ★ | ★ | ★ | ★ | ★★ | ★ | ★ | ★ | 9 |
| Barao 2017 ^(18)^ | ★ | ★ | ★ | ★ | ★★ | ★ |  | ★ | 8 |
| Maurício 2018 ^(23)^ | ★ | ★ | ★ | ★ | ★ | ★ | — | ★ | 7 |
| Huang 2019 ^(24)^ | ★ | ★ | ★ | ★ | ★ | ★ | — | ★ | 8 |
| Gallois 2019 ^(12)^ |  | ★ | ★ | ★ | ★ | ★ | ★ | ★ | 7 |
| Tsai 2020 ^(25)^ | ★ | ★ | ★ | ★ | ★★ | ★ | — | ★ | 8 |
| Fang 2020 ^(13)^ | ★ | ★ | ★ | ★ | ★ |  | ★ | ★ | 7 |
| De Groot 2020 ^(14)^ |  | ★ | ★ | ★ | ★ | ★ | ★ |  | 6 |
| Chen 2021 ^(15)^ | ★ | ★ | ★ | ★ | ★★ | ★ | ★ | ★ | 9 |
| Findlay 2021 ^(16)^ | ★ | ★ | ★ | ★ | ★★ | ★ | ★ | ★ | 8 |
| Von Geldern 2021 ^(26)^ |  | ★ | ★ | ★ | ★ | ★ | ★ | ★ | 7 |
| Zhang 2021 ^(27)^ |  | ★ | ★ | ★ | ★★ | ★ | ★ | ★ | 8 |
| Nikniaz 2022 ^(28)^ | ★ | ★ | ★ | ★ | ★★ | ★ | ★ | ★ | 9 |
| Ruan 2022 ^(29)^ | ★ | ★ | ★ | ★ | ★★ | ★ | ★ | ★ | 9 |
| de Sousa 2022 ^(30)^ |  | ★ | ★ | ★ | ★★ | ★ | ★ | ★ | 8 |
| Argefa 2022 ^(31)^ | ★ | ★ | ★ | ★ | ★ | ★ |  |  | 6 |
| da Silva Couto 2023 ^(32)^ | ★ | ★ | ★ | ★ | ★★ | ★ | ★ | ★ | 9 |
